# Pitfalls and artifacts in the interpretation of oncologic PET/CT of the chest

**DOI:** 10.1590/0100-3984.2015.0194

**Published:** 2017

**Authors:** Gustavo de Souza Portes Meirelles, Julia Capobianco, Marco Antônio Condé de Oliveira

**Affiliations:** 1 MD, PhD, Postdoctoral work in PET/CT, Medical Coordinator of the Grupo Fleury, São Paulo, SP, Brazil.; 2 MD, Specialist in PET/CT, Radiologist for the Grupo Fleury, São Paulo, SP, Brazil.; 3 Nuclear Medicine Physician for the Grupo Fleury, São Paulo, SP, Brazil.

**Keywords:** Pitfalls, Chest, PET/CT, Oncology

## Abstract

PET/CT is widely used for the evaluation of patients with thoracic malignancies. Although the levels of ^18^F-fluorodeoxyglucose (FDG) uptake are usually high in neoplastic diseases, they can also be physiological, due to artifacts. In addition, FDG uptake can occur in benign conditions such as infectious, inflammatory, and iatrogenic lesions. Furthermore, some malignant tumors, such as adenocarcinoma in situ (formerly known as bronchoalveolar carcinoma) and carcinoid tumors, may not show FDG uptake. Here, we illustrate the main pitfalls and artifacts in the interpretation of the results of oncologic PET/CT of the chest, outlining strategies for avoiding misinterpretation.

## Abstract (pt)

PET/CT é amplamente utilizada para avaliação de pacientes com neoplasias torácicas. Altos níveis de captação de ^18^F-fluordesoxiglicose (FDG) são geralmente vistos em neoplasias, mas também podem ser fisiológicos, decorrentes de artefatos ou ocorrerem em condições benignas, como lesões infecciosas, inflamatórias e iatrogênicas. Por outro lado, alguns tumores malignos podem não captar FDG, como o adenocarcinoma in situ (anteriormente denominado de carcinoma bronquioloalveolar) e tumores carcinoides. Os autores ilustram as principais armadilhas e artefatos na interpretação dos exames torácicos de PET/CT oncológicos, com estratégias para evitar erros de interpretação.

## INTRODUCTION

Positron emission tomography/computed tomography (PET/CT) imaging has been the subject of a series of recent publications in the radiology literature in Brazil^(1-10)^. PET/CT is an integral part of the management of patients with thoracic neoplasms, improving staging, monitoring of therapy, and prognostic assessment. However, many artifacts and pitfalls can be seen on the examination, such as normal variants, physiological areas of ^18^F-fluorodeoxyglucose (FDG) uptake, acquisition or reconstruction artifacts, and false-positive or false-negative results.

The purpose of this essay was to describe and illustrate the main pitfalls and artifacts in the interpretation of oncologic PET/CT examinations of the chest and to present strategies for avoiding their ‘misinterpretation.

## PHYSIOLOGICAL FDG UPTAKE

**Figure 1:**
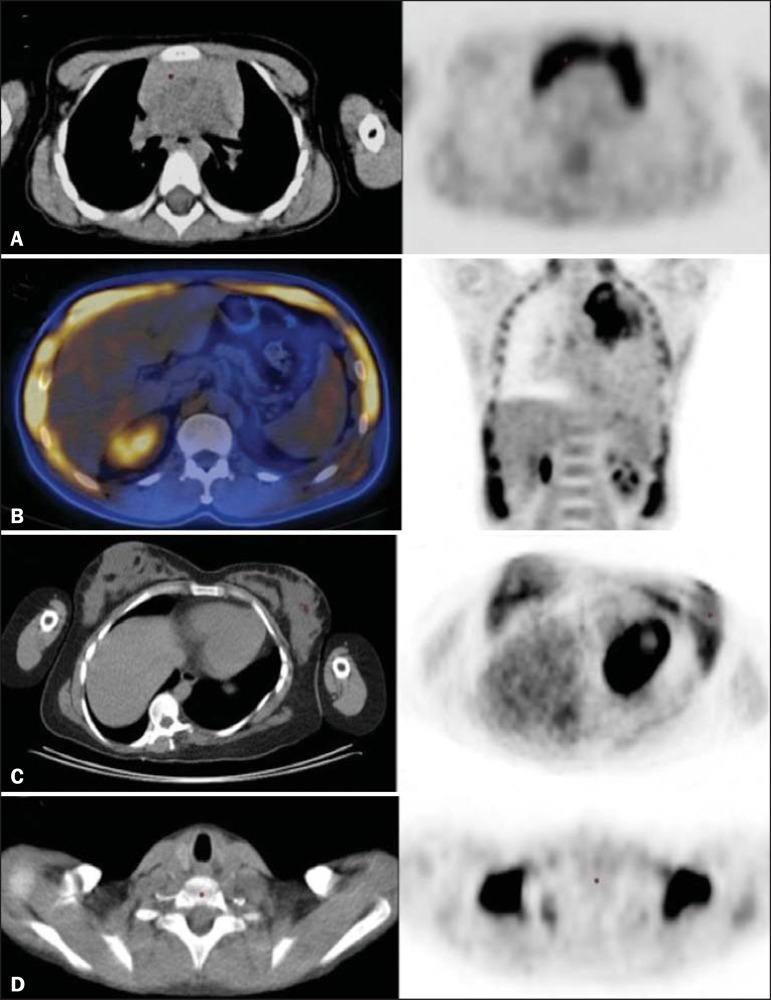
*Causes of physiological FDG uptake in the thorax.* **A:** A 6-year-old boy, with normal metabolic activity in the thymus. The strategy for differentiating between focal uptake in the anterior mediastinum and a lesion is to look at the normal aspect of the thymus on CT images. **B:** A 56-year-old man with lung cancer and a forceful cough during the PET/CT examination. FDG PET/CT shows increased metabolic activity in the thoracic muscles, due to vigorous coughing. **C:** A 31-year-old lactating woman, with normal FDG uptake in the breast. Lactation induces higher metabolic activity in the breasts, which should not be interpreted as disease. **D:** A 28-year-old man with testicular cancer. Multiple areas of FDG uptake in the supraclavicular regions and in the upper mediastinum, consistent with increased metabolic activity in brown fat, which regulates body weight and temperature and can be activated by satiety or cold environments.

FDG uptake in PET/CT can be seen in active tissues with high glucose metabolism. High physiological uptake of FDG typically occurs in the brain, lymphoid tissue, liver, spleen, kidneys, and urinary tract^(11)^. In the thorax, normal metabolic activity can be seen in the myocardium^(12)^, great vessels, esophagus, thymus, breast (especially of lactating females), bone marrow, muscles, and brown fat (Figure 1)^(13)^.

## FALSE-POSITIVE FDG UPTAKE

False-positive FDG uptake can occur in infections, inflammatory lesions, benign tumors, and iatrogenic conditions such as surgical manipulation, pleurodesis, radiation therapy, and granulocyte-colony-stimulating factor administration, as well as after chemotherapy (Figures 2-10)^(13-17)^.

**Figure 2:**
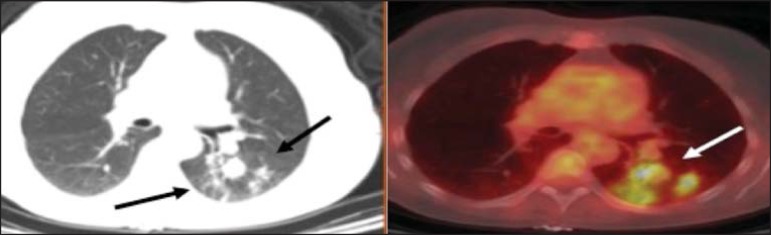
A 57-year-old female with cough, fever, and multiple centrilobular pulmonary nodules in the superior segment of the left lower lobe (arrows). PET/CT was performed and showed increased FDG uptake (arrow) in the lung nodules. A CT-guided pulmonary biopsy confirmed the diagnosis of pulmonary tuberculosis.

**Figure 3:**
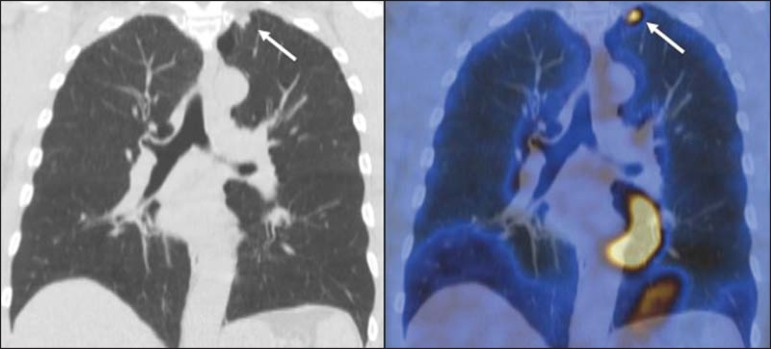
A 57-year-old man, current smoker, with non-Hodgkin lymphoma and a spiculated pulmonary nodule (arrows) in the left upper lobe, with high FDG uptake. Surgical resection confirmed the diagnosis of pulmonary cryptococcosis.

**Figure 4:**
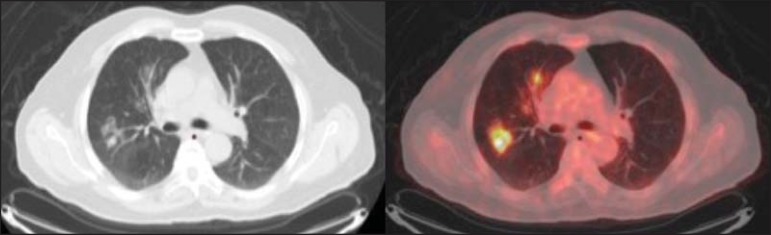
A 74-year-old man with esophageal cancer and multiple areas of ground-glass opacities with FDG uptake in the right lung, consistent with bacterial pneumonia.

**Figure 5:**
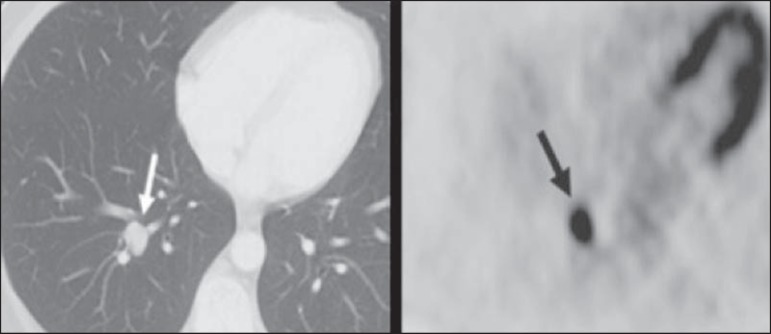
A 42-year-old man with a lobulated pulmonary nodule in the right lower lobe (arrows), indeterminate on CT. PET/CT was performed and showed increased metabolic activity (arrow) in the lung nodule, which was surgically resected and was found to be consistent with pulmonary meningioma.

**Figure 6:**
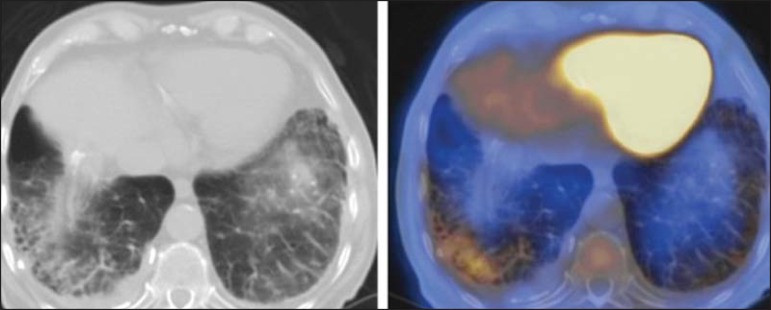
A 81-year-old man with an occupational history of asbestos exposure. PET/CT images demonstrate high metabolic activity in the left lower lobes, especially in the cortical regions. CT images depict interstitial lung abnormalities, with ground-glass opacities, reticulation, and mild bronchiolectasis.

**Figure 7:**
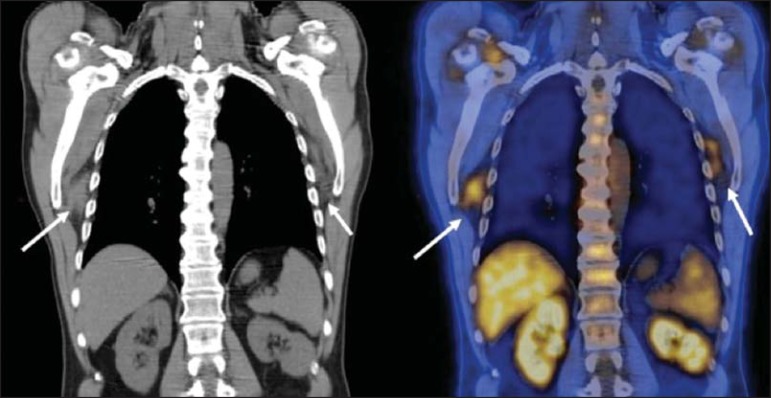
A 78-year-old woman submitted to PET/CT for the staging of a cervical carcinoma, with increased FDG uptake in bilateral subscapular masses (arrows), consistent with elastofibroma dorsi.

**Figure 8:**
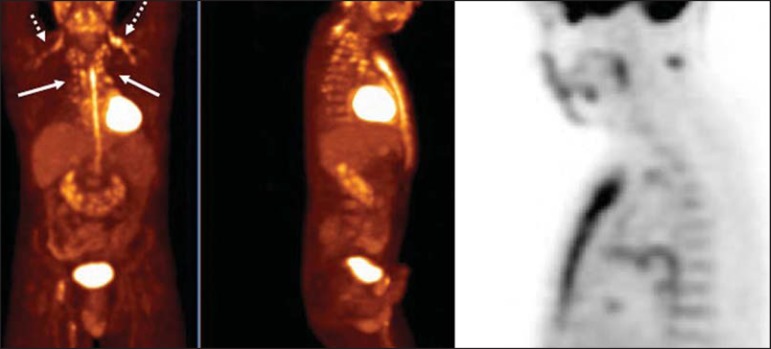
Postoperative PET/CT of a patient with history of cardiac tumor. Note the diffuse FDG uptake in the sternum, consistent with inflammatory changes induced by the surgical procedure (arrows). Also note the increased metabolic activity in areas of brown fat in the supraclavicular and paravertebral regions (dashed arrows).

**Figure 9:**
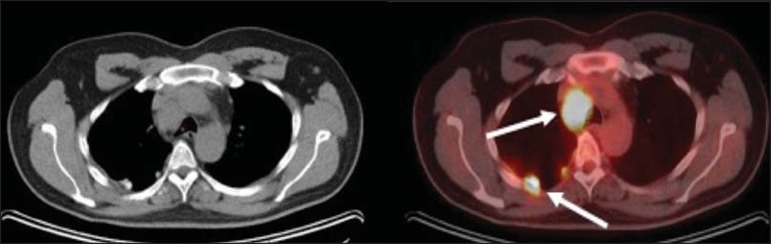
A 64-year-old woman submitted to PET/CT for the restaging of lung cancer. Multiple foci of FDG uptake are seen in the right pleura (arrows), consistent with an inflammatory process induced by pleurodesis. A careful analysis of CT images demonstrates the high-attenuation pleural thickening, characteristic of pleurodesis.

**Figure 10:**
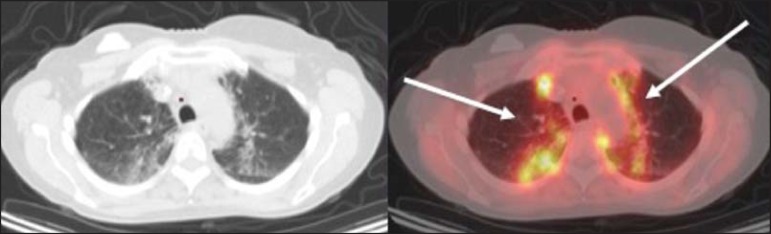
A 41-year-old woman with a history of Hodgkin lymphoma and paramediastinal opacities with FDG uptake (arrows), consistent with inflammatory lesions induced by radiation therapy.

## FALSE-NEGATIVE STUDIES

**Figure 11:**
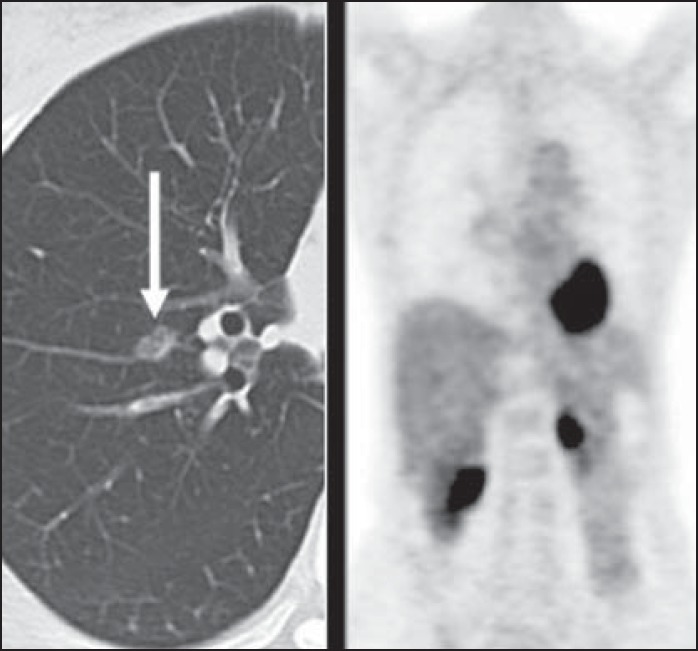
A 63-year-old male, current smoker, with a ground-glass nodule in the right lung (arrow), which had consistently increased in size over the years. PET/CT was performed and was negative. Surgical resection confirmed the diagnosis of adenocarcinoma *in situ* (formerly known as bronchoalveolar carcinoma). PET/CT is limited for these conditions and should not be used for small nodules with groundglass attenuation, due to the high pretest probability of false-negative results.

False-negative FDG uptake (Figure 11) can be seen in adenocarcinomas *in situ* (formerly known as bronchoalveolar carcinomas), carcinoid tumors^(18)^, ground-glass nodules, and small lesions^(19)^.

## ARTIFACTS

**Figure 12:**
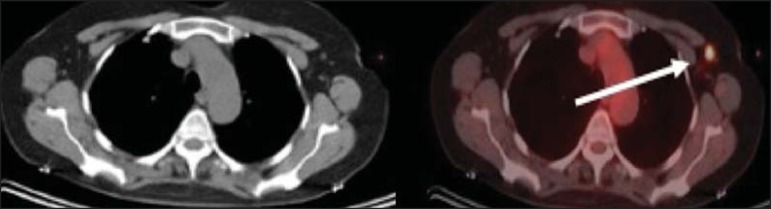
A 57-year-old woman with breast cancer and focal FDG uptake in a left axillary lymph node (arrow). That uptake is consistent with extravasation of FDG in the left arm, draining to the ipsilateral axillary region.

**Figure 13:**
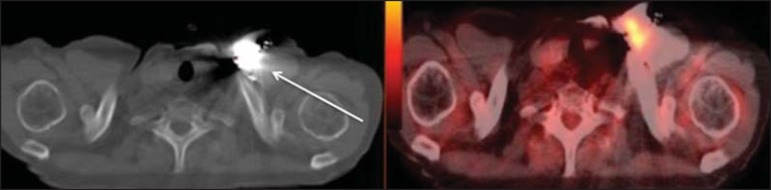
A 78-year-old man with a pacemaker in his left hemithorax (arrow). A PET attenuation-corrected image shows FDG uptake in the left hemithorax, consistent with an artifact induced by attenuation correction. In cases of uncertainty, the non-attenuation-corrected image can be used in order to avoid misinterpretation.

Artifacts (Figures 12 and 13) can be induced by PET attenuation correction, misregistration related to free breathing, truncation, FDG extravasation, and FDG embolism^(20)^.

## CONCLUSION

Awareness of normal FDG distribution, physiological FDG uptake, and their variants is mandatory for interpreting oncologic PET/CT examinations of the chest. False-positive and false-negative results can be avoided if the reader has knowledge of their main aspects and interprets the CT and PET findings carefully. When these points are well known by the radiologist, PET/CT is a powerful imaging technique for characterizing pulmonary lesions, providing accurate staging for lung neoplasms and contributing to better evaluation of the effectiveness of therapy and prognostic assessment.
